# Association of Malnutrition and High Bleeding Risk with Long-Term Prognosis in Patients with Acute Coronary Syndrome following Percutaneous Coronary Intervention

**DOI:** 10.3390/medicines10120062

**Published:** 2023-11-30

**Authors:** Hiromitsu Kataoka, Sayumi Suzuki, Yuichi Suzuki, Ryota Sato, Makoto Sano, Satoshi Mogi, Atsushi Sakamoto, Kenichiro Suwa, Yoshihisa Naruse, Hayato Ohtani, Masao Saotome, Mikihiro Shimizu, Keiichi Odagiri, Yuichiro Maekawa

**Affiliations:** 1 Division of Cardiology, Internal Medicine III, Hamamatsu University School of Medicine, Hamamatsu 431-3192, Japan; rlseg23@gmail.com (H.K.); s.sayumi@hama-med.ac.jp (S.S.); yuichi900921@gmail.com (Y.S.); rsato@hama-med.ac.jp (R.S.); makosano@hama-med.ac.jp (M.S.); s.mogi@hama-med.ac.jp (S.M.); asakamo@hama-med.ac.jp (A.S.); k-suwa@hama-med.ac.jp (K.S.); ynaruse@hama-med.ac.jp (Y.N.); ohtani@hama-med.ac.jp (H.O.); msaotome@hama-med.ac.jp (M.S.); 2 Center for Clinical Research, Hamamatsu University Hospital, Hamamatsu 431-3192, Japan; shimizu.mi@hama-med.ac.jp (M.S.); kodagiri@hama-med.ac.jp (K.O.)

**Keywords:** acute coronary syndrome, malnutrition, percutaneous coronary intervention, high bleeding risk

## Abstract

Background: Malnutrition in cardiovascular disease is associated with poor prognosis, especially in patients with heart failure and acute coronary syndrome (ACS). High bleeding risk is also linked to coronary artery disease prognosis, including ACS. However, whether the extent of malnutrition and high bleeding risk have a cumulative impact on the long-term prognosis of patients with ACS who undergo percutaneous coronary intervention remains unclear. Methods: We analyzed 275 patients with ACS treated with percutaneous coronary intervention. The Controlling Nutritional Status score and Japanese version of the Academic Research Consortium for High Bleeding Risk criteria (J-HBR) were retrospectively evaluated. The primary and secondary outcomes were adjusted using the inverse probability treatment weighting method. Results: The prevalence of moderate or severe malnutrition in this cohort was 16%. Kaplan–Meier analysis showed a significantly higher incidence of major adverse cardiovascular and cerebrovascular events in patients who were moderately or severely malnourished than in those who were not. Notably, the incidence of these major events was similar between severely malnourished patients with J-HBR and those without. Conclusion: Moderate or severe malnutrition has a significant impact on the long-term prognosis of patients with ACS who undergo percutaneous coronary intervention.

## 1. Introduction

Elderly patients have unique problems, one of which is malnutrition [1]. The implementation of nutritional checks is used to evaluate prognosis for chronic heart failure [2,3] but this is not so commonly applied to those with coronary artery disease. The Controlling Nutritional Status (CONUT) score has been linked to the prognosis of patients with non-ST elevation myocardial infarction (MI) undergoing percutaneous coronary intervention (PCI) [4]. In patients with acute coronary syndrome (ACS) who undergo PCI with high bleeding risk (HBR), prognosis is poor due to increased bleeding events [5,6,7,8]. The latest guidelines from the Japanese Society of Cardiology added heart failure, low body weight, peripheral artery disease and frailty to the Academic Research Consortium for High Bleeding Risk (ARC-HBR) criteria as the Japanese version of the ARC-HBR (J-HBR) [9].

Although many older patients with ACS meet the J-HBR criteria in addition to malnutrition, the prognosis details of patients with malnutrition, especially moderate or severe malnutrition, and of those meeting the J-HBR criteria, are unknown. This study aimed to determine whether moderate and severe malnutrition meeting the J-HBR criteria in patients with ACS who underwent PCI has an impact on long-term prognosis.

## 2. Materials and Methods

### 2.1. Patient Population

We retrospectively examined 275 consecutive patients who underwent PCI for ACS with stenting. These patients were admitted to Hamamatsu University Hospital between January 2019 and March 2022. This study included patients with medical records that had necessary information to determine malnutrition using the CONUT scoring system and those with 1-year follow-up clinical assessments. Patients who lacked laboratory data, including total lymphocyte counts, serum albumin levels, and total cholesterol levels and patients undergoing hemodialysis were excluded. This study was performed in accordance with the Declaration of Helsinki and approved by the Institutional Review Board of Hamamatsu University School of Medicine (approval number: 23–128), with a waiver for informed consent.

### 2.2. Percutaneous Coronary Intervention

Coronary interventions were performed in accordance with standard techniques and international guidelines [10]. Before beginning the procedure, all patients were given 200 mg aspirin and either 300 mg clopidogrel or 20 mg prasugrel orally. All patients received a bolus injection of heparin (5000 IU), and the activated coagulation time was checked every 30 min and maintained at >250 s using additional boluses of heparin. Dual antiplatelet therapy with 100 mg/day aspirin and either 75 mg/day clopidogrel or 3.75 mg/day prasugrel was given after PCI. Coronary angiography in all patients was performed using a 5-Fr or 6-Fr catheter via the radial approach. Stent deployment was performed according to conventional methods using a 6-Fr guiding catheter, a 0.014-inch guidewire, and a monorail balloon catheter. All patients were evaluated via intravascular ultrasound or optical coherence tomography.

### 2.3. Clinical Assessments

Medical records, including medical history, physical examination, laboratory tests, 12-lead electrocardiogram, and, when available, echocardiographic findings, were carefully reviewed. The following data were obtained: age; sex; coronary risk factors (including cigarette smoking, hypertension, dyslipidemia, diabetes mellitus, and family history of premature coronary artery disease, defined as MI or sudden death in a first-degree relative, male younger than 55 years or female younger than 65 years); and concomitant medications before and after hospitalization (including anti-platelets, beta-blockers, angiotensin-converting enzyme inhibitors, angiotensin receptor blockers, statins, calcium channel blockers, and anti-coagulants). Follow-up data were obtained through direct contact at an outpatient clinic, via a telephone interview, or through a review of the medical records of surviving patients.

### 2.4. Nutritional Indices

The CONUT score was used to determine the patients’ nutritional status [11,12]. The CONUT scoring system requires total lymphocyte counts, serum albumin, and total cholesterol levels and has the following scores: 0–1, normal nutritional status; 2–4, mild risk; 5–8, moderate risk; and 9–12, severe risk of malnutrition.

### 2.5. Japanese Version of the ARC-HBR Criteria

The J-HBR criteria have been proposed through consensus of the Working Group of the Guidelines in the Japanese Circulation Society [9]. The J-HBR criteria include the Japanese-specific major criteria such as heart failure, low body weight, peripheral artery disease, frailty, and the minor ARC-HBR criteria. Patients were considered to have HBR if they met at least one major criterion or two minor J-HBR criteria. Therefore, patients with at least one major criterion such as severe chronic kidney disease (CKD), thrombocytopenia, severe anemia, liver cirrhosis, prior hemorrhagic stroke, active malignancy, anticoagulation, heart failure, low body weight, peripheral artery disease, and frailty, and those with two or more minor criteria, such as age ≥ 75 years, mild anemia, prior ischemic stroke, prior bleeding, and moderate CKD, were classified as the J-HBR (+) group. Patients who met only one minor criterion or no criteria were classified as the J-HBR (−) group.

### 2.6. Primary and Secondary Outcomes and Exploratory Analysis

The primary outcome was a major adverse cardiovascular and cerebrovascular event (MACCE) composite of all-cause death, including cardiovascular death, MI, stroke, stent thrombosis, and target vessel failure (TVF) at the 1-year follow-up. TVF incidence was evaluated at the 1-year follow-up by coronary angiography or cardiac computed tomography. The secondary outcome was all-cause mortality. An exploratory analysis was performed to clarify the association between moderate or severe malnutrition and J-HBR in patients with ACS following PCI.

### 2.7. Statistical Analysis

Continuous variables are expressed as mean ± standard deviation, and categorical variables are expressed as counts with percentages. Baseline, procedural, and medication variables were compared between patients with normal or mild malnutrition and those with moderate or severe malnutrition using Student’s *t*-tests, chi-square tests, or Fisher’s exact tests, as appropriate. To account for the non-randomized study design and to reduce the imbalance in baseline characteristics and the effect of a potential selection bias, an inverse probability treatment weighting (IPTW) analysis was performed, adjusted for variables selected based on clinical significance. The selected variables were age, diabetes, CKD, and history of heart failure. Patients were censored at the time of an event or at the end of the planned 1-year follow-up, depending on which occurred first. We plotted Kaplan–Meier curves using overall survival and MACCE data after IPTW. Differences between groups were assessed using the log-rank test. All statistical analyses were performed using SPSS version 25 (IBM Corp., Armonk, NY, USA). A *p*-value < 0.05 was considered significant in all analyses.

## 3. Results

### 3.1. Baseline and Procedural Characteristics

**Figure 1:**
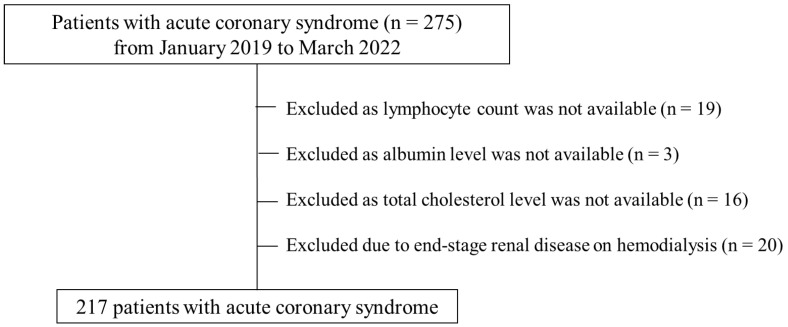
CONSORT diagram.

Of the 275 patients with ACS who underwent PCI, 38 lacked lymphocyte count, serum albumin, or total cholesterol levels, and 20 were undergoing hemodialysis; therefore, a total of 217 patients fulfilled the eligibility criteria and were included in the final analysis (Figure 1). Those who were included in the final analysis were grouped into two groups according to malnutritional severity based on the CONUT score; the normal or mild malnutrition group included patients with CONUT scores of 0–4, and the moderate or severe malnutrition group included patients with CONUT scores of 5–12. Baseline clinical characteristics after the IPTW analysis of the 217 patients are shown in Table 1. Table S1 shows the baseline clinical characteristics before the IPTW analysis. The moderate or severe malnutrition group had lower total lymphocyte counts, total cholesterol levels, and serum albumin levels. There was a significantly lower rate of thrombolysis in myocardial infarction grade 3 in the moderate or severe malnutrition group compared to that in the normal or mild malnutrition group. The incidence of complications such as coronary perforation and BARC 3a or 5 bleeding did not differ between the two groups (Table 2).

### 3.2. Nutritional Status of Patients with ACS

**Figure 2:**
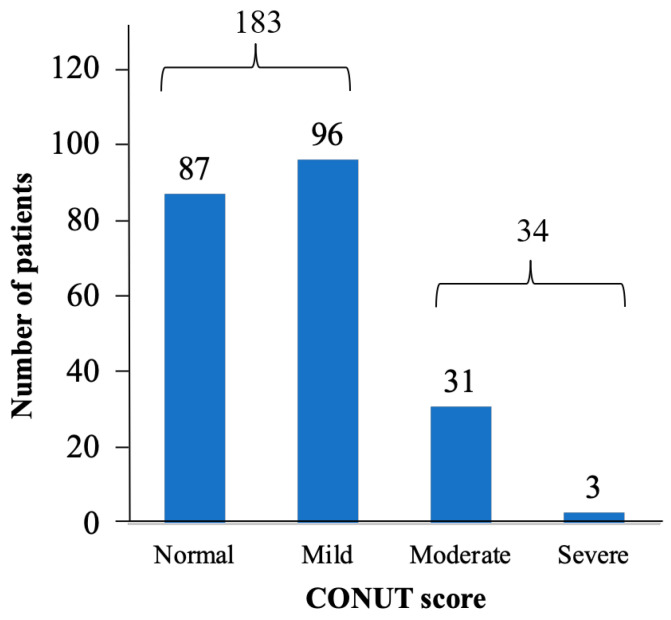
Nutritional status as determined by CONUT score for all patients.

The CONUT score was used to screen and evaluate malnutrition severity in the normal or mild (*n* = 183, 84.3%) and moderate or severe (*n* = 34, 15.7%) malnutrition groups (Figure 2).

### 3.3. Long-Term Clinical Outcomes

**Figure 3:**
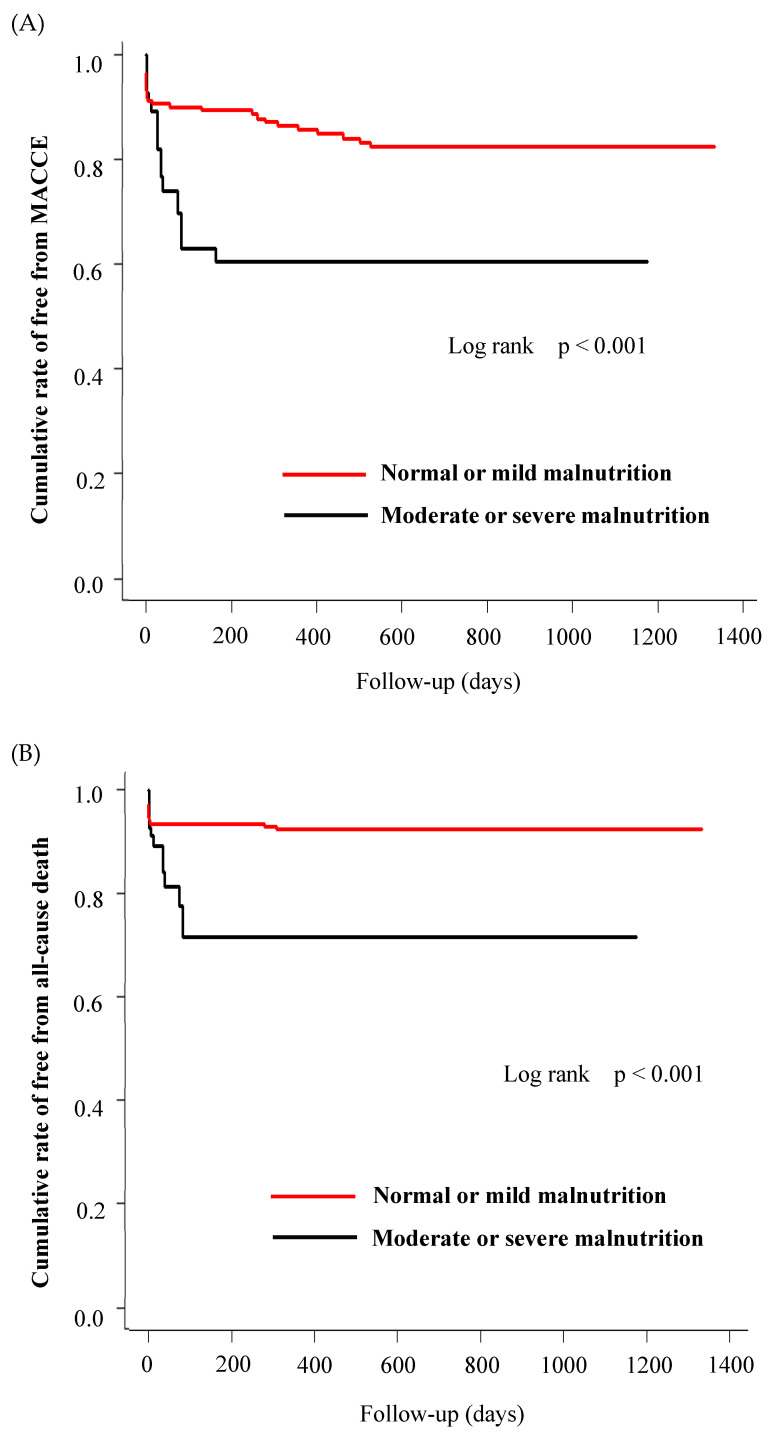
Kaplan–Meier survival curves for normal or mild malnutrition vs. moderate or severe malnutrition in ACS patients following PCI for (**A**) major adverse cardiovascular and cerebrovascular events (MACCE) and (**B**) all-cause death after inverse probability treatment weighting analysis.

All patients were followed up for a mean duration of 551.2 days after hospital discharge. Long-term clinical outcomes for 1 year after IPTW analysis are summarized in Table 3. The primary end points, namely all-cause death, including cardiovascular death; MI; stroke; stent thrombosis; or TVF, occurred in 12.1% of the normal or mild malnutrition group versus 37.1% of the moderate or severe malnutrition group (Table 3, Figure 3A). The secondary end point, all-cause death, occurred in 4.8% of the normal or mild malnutrition group and in 28.2% of the moderate or severe malnutrition group (Table 3, Figure 3B). The incidences of MI, Stroke, TVF, and stent thrombosis did not differ between the two groups (Table 3).

### 3.4. Association between Nutrition Status and J-HBR Score

**Figure 4:**
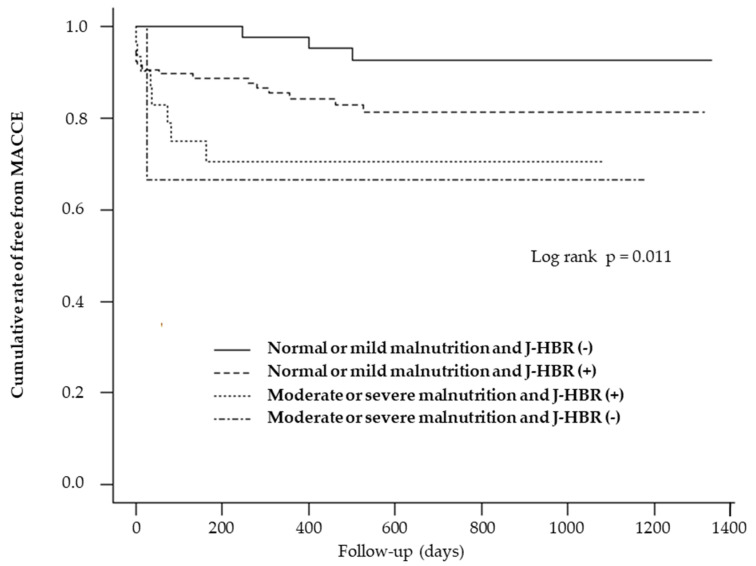
Kaplan−Meier analysis for cumulative major adverse cardiovascular and cerebrovascular events (MACCE) rate analyses were stratified by nutritional categories and the Japanese version of the Academic Research Consortium for High Bleeding Risk criteria (J-HBR).

The Kaplan−Meier curves demonstrated that MACCE significantly differed among patients with normal or mild malnutrition plus J-HBR, normal or mild malnutrition only, moderate or severe malnutrition plus J-HBR, and moderate or severe malnutrition only (*p* = 0.001). MACCE incidence was similar between severely malnourished patients with J-HBR and those without J-HBR (Figure 4).

## 4. Discussion

This study investigated whether moderate or severe malnutrition and meeting J-HBR criteria in patients with ACS who underwent PCI have an impact on long-term prognosis. The main findings of this study showed that patients with ACS and moderate or severe malnutrition had worse long-term clinical outcomes than those with normal or mild malnutrition; J-HBR was not associated with long-term clinical outcomes in patients with moderate or severe malnutrition.

In this study, patients with moderate and severe malnutrition had more CKD than those with normal or mild malnutrition (Table S1), consistent with the findings of a previous study [12]. However, previous studies on the association between malnutrition and ACS prognosis included relatively young patients in their late 50 s and 60 s [13,14]. Meanwhile, the average age of the patients in this study was approximately 70 years, which reflects the age of many Japanese patients undergoing PCI [15,16].

The J-HBR is a bleeding risk criterion proposed in the guidelines established by the Japanese Circulation Society [9] and is frequently used to determine the duration of antithrombotic therapy after PCI. Although it is easy to conclude that malnourished patients are at higher risk of bleeding, and several papers have demonstrated this, the relationship between malnutrition and J-HBR, whether additive or synergistic, has been unclear in patients with ACS undergoing PCI. In this study, we compared the MACCE incidence in patients with normal or mild malnutrition plus J-HBR, normal or mild malnutrition only, moderate or severe malnutrition plus J-HBR, and moderate or severe malnutrition only. Surprisingly, MACCE rates were similar between moderately and severely malnourished patients with J-HBR and those without J-HBR. The following are possible reasons: about a half of patients undergone PCI in Japan have J-HBR [17], and many of the conditions included in the J-HBR criteria are related to malnutrition, such as advanced age, low body weight, CKD, and heart failure. Therefore, the J-HBR criteria themselves may have had little impact on the long-term prognosis of patients with ACS after PCI, independent of malnutrition.

The mechanisms underlying the prognostic role of CONUT scoring in ACS following PCI have not been thoroughly explored. The CONUT score is calculated from lymphocyte counts, total cholesterol, and albumin levels. Baseline albumin level can be used as a biomarker to indicate a patient’s overall nutritional and immune status [18]. Low serum albumin levels resulting from inflammation-induced capillary leakage or disease-related anorexia during acute illness are associated with poor outcomes [19]. Absolute lymphocyte count can indicate the host’s systemic immune status. In patients with ACS following PCI, a lower lymphocyte count is significantly associated with the incidence of stent thrombosis [20]. The administration of antiplatelet therapy is also required in malnourished patients with ACS, which leads to increased bleeding [21]. Taken together, bleeding and thrombogenicity result from malnutrition, which causes increased all-cause mortality in patients with ACS after PCI.

It has been reported that nutritional status should be assessed during heart failure treatment to predict heart failure prognosis [22,23]. Various studies have also shown that improving the nutritional status of heart failure patients can improve prognosis, prevent disease deterioration, and decrease patient mortality [24,25]. We have reported that non-dietitians use “objective nutritional indicators” less frequently than dietitians in providing nutritional guidance to patients with heart failure [26]. Considering that not every facility has a registered dietitian, it is possible that more accurate and consistent patient assessments could be achieved if each profession utilized objective measures. Although this study has limitations because it is a retrospective study, it reaffirms the importance of assessing nutritional status and intervening using the CONUT score, an objective measure, in ACS cases. The specific method of nutritional intervention is to formulate a nutrition plan based on nutritional information and the cause of anorexia and to conduct nutritional management. First, depending on the dietary intake status, adjustments are made to the amount of food intake and to dietary preferences. Individualized dietary support is provided, such as the adjustment of the appropriate diet. In addition, nutritional education, such as explaining the necessity of eating and the significance of nutritional intake in order to maintain and improve nutritional status, has been practiced in patients with heart failure [27], and the same approach is considered effective in patients with ACS. We believe that the results of this study are important for implementing such an approach.

In addition, while it is difficult to intervene in many of the J-HBR criteria regardless of treatment, two of them, underweight and frailty, are closely related to malnutrition. The results of this study indicate that more severe malnutrition may be associated with a long-term prognosis than high bleeding risk and strongly emphasize the need for nutritional intervention. In the subjects of this study, patients with an average age of 70 years, a different nutritional approach is needed than in the so-called middle-aged obese patients [28], and one of the approaches is a multidisciplinary approach consisting of physicians, nurses, dietitians, and pharmacists. This approach is expected to improve nutritional status. It is easy to imagine that this approach will result in the improvement of sarcopenia, a phenotype of frailty and frailty that is well known to have a significant impact on the prognosis of cardiovascular diseases. We demonstrated that more severe malnutrition had a great impact on the long-term prognosis of patients with ACS who underwent PCI. This result is not new, but few reports have examined the long-term prognosis of J-HBR in combination with nutritional status, which is a factor that cannot be ignored for the Japanese population, who are at a potentially high risk of bleeding. As shown in Table 3, the incidence of MI, stroke, TVF, and stent thrombosis did not differ between the two groups, but there was a significant difference in all-cause mortality. This is consistent with the results of several previous studies [29,30].

To conclude, nutritional status is very important in predicting the MACCE rate at the 1-year follow-up in patients undergoing PCI for ACS. Evaluating both nutritional status and bleeding risk in patients with ACS is meaningful, but malnutrition appears to have greater significance than bleeding risk in terms of long-term survival.

### Limitations

This study has a few limitations. First, this study employed only one scoring system related to nutritional status. Nevertheless, the CONUT score is one of the most reliable objective nutritional scores. Second, the sample size was too small for any conclusions to be drawn, but we were able to collect more detailed data compared with other studies. Third, a longer follow-up is needed to evaluate the association between nutritional status and bleeding score. Furthermore, the present cohort was based on a limited number of patients at a single institution; thus, future validation is needed.

## Figures and Tables

**Table 1:** Baseline characteristics after inverse probability treatment weighting analysis.

Baseline Characteristics	Normal or Mild Malnutrition (*n* = 183)	Moderate or Severe Malnutrition (*n* = 34)	SMD	*p*-Value
Age, years	71.6 (11.1)	72.3 (9.9)	0.065	0.757
Male	79.2	75.3	0.093	0.650
BMI, kg/m^2^	24.3 (4.3)	22.6 (4.4)	0.374	0.113
Hypertension	66.9	55.9	0.228	0.307
DM	43.4	43.0	0.029	0.892
Dyslipidemia	66.7	51.2	0.320	0.148
CKD	34.2	33.3	0.018	0.926
Smoking	65.4	49.0	0.337	0.130
Past history of HF	12.1	10.6	0.047	0.779
Family history of CAD	2.9	1.5	0.097	0.532
Previous MI	17.4	27.3	0.239	0.271
Previous PCI	28.1	30.1	0.045	0.841
Previous AF/AFL	12.5	23.7	0.295	0.166
Previous PAD	10.6	13.1	0.075	0.709
CONUT	1.74 (1.29)	5.94 (1.25)	3.293	<0.001
J-HBR (+)	66.6	83.0	0.384	0.168
ACS classification				
STEMI	56.1	67.2	0.231	0.283
NSTEMI	17.9	14.8	0.085	0.652
UA	26.0	18.0	0.195	0.390
Access site				
Radial approach	90.6	84.7	0.180	0.374
Medications				
Aspirin	93.2	83.4	0.311	0.121
Clopidogrel	24.8	32.1	0.162	0.432
Prasugrel	70.5	54.6	0.334	0.127
ACE-inhibitor/ARBs	70.2	36.0	0.730	0.001
β blockers	67.5	55.6	0.247	0.270
Statin	95.8	81.8	0.456	0.006
Ca channel blockers	29.4	10.2	0.495	0.012
DOAC	9.2	17.9	0.254	0.215
Warfarin	4.5	1.3	0.193	0.211
Echocardiographic data				
LVEF, %	49.6 (12.4)	44.5 (14.3)	0.379	0.160
Laboratory data				
White blood cell count, /μl	7809.7 (2837.6)	8176.1 (3094.4)	0.123	0.566
Total lymphocyte count, /μl	1707.8 (732.8)	1146.1 (775.6)	0.744	0.003
Triglyceride, mg/dL	131.0 (98.8)	78.1 (57.4)	0.655	<0.001
Total cholesterol, mg/dL	174.3 (44.5)	136.6 (45.9)	0.834	<0.001
HDL-C, mg/dL	45.0 (12.2)	38.1 (15.1)	0.503	0.045
LDL-C, mg/dL	102.6 (37.4)	78.0 (30.5)	0.720	<0.001
Albumin, mg/dL	4.0 (0.4)	3.1 (0.4)	2.136	<0.001
Total protein, mg/dL	7.1 (4.5)	6.0 (0.9)	0.349	0.002

Data are mean (standard deviation) or %. Abbreviations: SMD, standardized mean differences; BMI, body mass index; DM, diabetes mellitus; CKD, chronic kidney disease; HF, heart failure; CAD, coronary artery disease; MI, myocardial infarction; PCI, percutaneous coronary intervention; AF/AFL, atrial fibrillation/atrial flutter; PAD, peripheral artery disease; CONUT, Controlling Nutritional Status; J-HBR, J-high bleeding risk; ACS, acute coronary syndrome; STEMI, ST elevated myocardial infarction; NSTEMI, non-ST elevation myocardial infarction; UA, unstable angina; ACE, angiotensin-converting enzyme; ARBs, angiotensin II receptor blockers; DOAC, direct oral anti-coagulants; LVEF, left ventricular ejection fraction; HDL-C, high density lipoprotein cholesterol; LDL-C, low density lipoprotein cholesterol.

**Table 2:** Procedural characteristics and complications after inverse probability treatment weighting analysis.

Procedural Characteristics	Normal or Mild Malnutrition (*n* = 183)	Moderate or Severe Malnutrition (*n* = 34)	*p*-Value
TIMI grade 3	94.3	75.4	0.002
Complication			
Coronary perforation,	0	0	1
BARC 3a or 5 Bleeding	9.9	19.7	0.192

Data are %. Abbreviations: TIMI, thrombolysis in myocardial infarction; BARC, bleeding academic research consortium.

**Table 3:** Long-term clinical outcomes after inverse probability treatment weighting analysis.

Clinical Outcomes	Normal or Mild Malnutrition (*n* = 183)	Moderate or Severe Malnutrition (*n* = 34)	*p*-Value
MACCE	12.1	37.1	0.003
All-cause death	4.8	28.2	<0.001
Myocardial infarction	0.5	0	0.668
Stroke	1.8	6.7	0.219
TVF	4.5	2.1	0.475
Stent thrombosis	1.0	0	0.545

Data are %. Abbreviations: MACCE, major adverse cardiovascular and cerebrovascular events; TVF, target vessel failure.

## Data Availability

The data presented in this study are available on request from the corresponding author.

## Supplementary Materials

The following supporting information can be downloaded at: https://www.mdpi.com/article/10.3390/medicines10120062/s1; Table S1: Baseline Characteristics before inverse probability treatment weighting analysis.
