# Supplementary material for: Association of Malnutrition and High Bleeding Risk with Long-Term Prognosis in Patients with Acute Coronary Syndrome following Percutaneous Coronary Intervention
Source: Medicines (Basel). 2023 Nov 30;10(12):62. doi: 10.3390/medicines10120062 (PMC10744455; doi:10.3390/medicines10120062)
Supplement: Supplementary file 1 [file medicines-10-00062-s001.zip › medicines-2622546-supplementary.pdf]

Supplementary Table S1

Baseline Characteristics before inverse probability treatment weighting analysis.

	Normal or mild malnutrition (n=183)	Moderate or severe malnutrition (n=34)	P value
Age, years	70.7 (11.0)	75.6 (9.4)	0.016
Male	80.3	73.5	0.369
BMI, kg/m2	24.2 (4.2)	22.9 (4.1)	0.124
Hypertension	66.1	67.6	0.863
DM	39.8	61.7	0.018
Dyslipidemia	67.7	55.8	0.18
CKD	29.5	58.8	<0.001
Smoking	66.6	50.0	0.063
Past history of HF	10.3	20.5	0.144
Family history of CAD	3.2	2.9	1
Previous MI	16.3	29.4	0.072
Previous PCI	26.2	32.3	0.461
Previous AF/AFL	11.4	20.5	0.164
Previous PAD	10.3	17.6	0.242
CONU	1.2	1.5	<0.001
J-HBR (+)	64.4	91.1	0.002
ACS classification			
STEMI	56.8	55.8	0.918
NSTEMI	17.4	23.5	0.404
UA	25.6	20.5	0.528
Access site			
Radial approach	90.7	79.4	0.071
Medications			
Aspirin	93.9	88.2	0.263
Clopidogrel	22.9	44.1	0.01
Prasugrel, n (%)	72.1	50.0	0.011
ACE-inhibitor/ARBs	70.4	35.2	<0.001
β blockers	66.6	61.7	0.58
Statin	95.6	82.3	0.011
Ca channel blockers	28.4	14.7	0.095
DOAC	8.7	14.7	0.339

Warfarin	4.3	2.9	1
Echocardiographic data			
LVEF, %	50.3 (11.3)	46.2 (14.1)	0.127
Laboratory data			
White blood cell count, /μl	7840.9 (2887.1)	8410.5 (3416.6)	0.306
Total lymphocyte count, /μl	1722.3 (731.5)	1121.5 (640.2)	<0.001
Triglyceride, mg/dL	131.1 (98.5)	86.7 (70.0)	0.013
Total cholesterol, mg/dL	175.5 (44.9)	136.9 (46.2)	<0.001
HDL-C, mg/dL	45.1 (12.3)	37.9 (13.4)	0.002
LDL-C, mg/dL	103.7 (37.8)	76.5 (31.1)	<0.001
Albumin, mg/dL	4.0 (0.4)	3.0 (0.4)	<0.001
Total protein, mg/dL	7.1 (4.7)	5.8 (0.8)	0.135

Data are mean (standard deviation) or %. Abbreviations: BMI, body mass index; DM, diabetes mellitus; CKD, chronic kidney disease; HF, heart failure; CAD, coronary artery disease; MI, myocardial infarction; PCI, percutaneous coronary intervention; AF/AFL, atrial fibrillation/atrial flutter; PAD, peripheral artery disease; CONUT, controlling nutritional status; J-HBR, J-high bleeding risk; ACS, acute coronary syndrome; STEMI, ST elevated myocardial infarction; NSTEMI, non-ST elevation myocardial infarction; UA, unstable angina; ACE, angiotensin-converting enzyme; ARBs, angiotensin II receptor blockers; DOAC, direct oral anti-coagulants; LVEF, left ventricular ejection fraction; HDL-C, high density lipoprotein cholesterol; LDL-C, low density lipoprotein cholesterol
